# Pathophysiology of primary hypertension in children and adolescents

**DOI:** 10.1007/s00467-023-06142-2

**Published:** 2023-09-13

**Authors:** Mieczysław Litwin

**Affiliations:** Department of Nephrology and Arterial Hypertension, The Children’s Memorial Health Institute, Warsaw, Poland

**Keywords:** Primary hypertension, Pathophysiology, Children, Pathogenesis, Page mosaic

## Abstract

The progress in research on the physiology of the cardiovascular system made in the last 100 years allowed for the development of the pathogenesis not only of secondary forms of hypertension but also of primary hypertension. The main determinants of blood pressure are described by the relationship between stroke volume, heart rate, peripheral resistance, and arterial stiffness. The theories developed by Guyton and Folkow describe the importance of the volume factor and total peripheral resistance. However, none of them fully presents the pathogenesis of essential hypertension. The multifactorial model of primary hypertension pathogenesis developed by Irving Page in the 1940s, called Page's mosaic, covers most of the pathophysiological phenomena observed in essential hypertension. The most important pathophysiological phenomena included in Page's mosaic form a network of interconnected “nodes”. New discoveries both from experimental and clinical studies made in recent decades have allowed the original Page mosaic to be modified and the addition of new pathophysiological nodes. Most of the clinical studies confirming the validity of the multifactorial pathogenesis of primary hypertension concern adults. However, hypertension develops in childhood and is even perinatally programmed. Therefore, the next nodes in Page’s mosaic should be age and perinatal factors. This article presents data from pediatric clinical trials describing the most important pathophysiological processes associated with the development of essential hypertension in children and adolescents.

## Introduction

### Basics of physiology of arterial blood pressure regulation

**Fig. 1:**
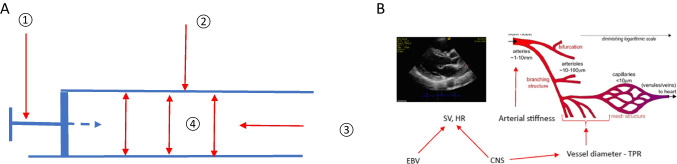
**A** Schematic presentation of fluid flow in the pipe: (1) pushing force; (2) pipe stiffness; (3) fluid viscosity and volume; (4) the pressure exerted by the fluid on the walls of the pipe. Unlike the arterial system, the resistance to flow in a pipe is the viscosity and volume of the fluid (3). **B** Schematic presentation of circulatory system and main determinants of blood pressure. The pulse wave causes an increase in SBP from the aorta, through the elastic arteries, to the muscular arteries. Despite the higher SBP in these segments of the arterial system, the mean arterial pressure is lower in them than in the aorta, which ensures the maintenance of blood flow. In the resistance arteries (arterioles), there is a large decrease in arterial pressure and a gradual decrease in the difference between SBP and DBP. Adapted from Keelan J, Hague JP (2021) Sci Rep 11:5408. CNS, central nervous system; DBP, diastolic blood pressure; EBV, effective blood volume; HR, heart rate; SBP, systolic blood pressure; SV, stroke volume; TPR, total peripheral resistance

Appropriate arterial blood pressure (BP) adjusts the perfusion of vital organs and tissues, oxygen supply, and removal of metabolic products depending on current metabolic needs. The basic principles characterizing arterial pressure determinants are the laws of physics that describe the flow of fluid in pipes. The pressure exerted by a fluid flowing in a pipe is a derivative of the force pushing the fluid, the volume of the fluid, the resistance it must overcome due to fluid viscosity, the pipe diameter, and elasticity/stiffness of the pipe wall. In the case of the circulatory system, the volume of blood ejected from the left ventricle (LV) is the stroke volume (SV), which depends, on the one hand, on the volume of circulating blood and, on the other hand, on the contraction force of LV, the stiffness of conductive vessels, and total peripheral resistance (TPR) (Fig. 1). The main determinant of volume is sodium chloride (NaCl) and the systems regulating salt and water homeostasis. The circulatory system is a system of tubes branching off and gradually decreasing in diameter from 2 cm at the level of the aorta down to less than 10 μm. The diameters of successive generations of arteries gradually decrease, and the resistance increases with the subsequent division of the artery. Arterioles, which are 10–100 µm in diameter and innervated and surrounded by smooth muscle cells, are the main element of the circulatory system that generate TPR. However, resistance to arterial flow is generated also by every arterial division. The microcirculation system consists of the above mentioned arterioles, which are interconnected with numerous anastomoses, and creates a network structure. The wall tension of the resistance vessels and their diameter are dynamically regulated by the current metabolic demand and BP. The reaction to excessively high pressure of the flowing blood is the contraction of the smooth muscle cells of the vessel wall and decreased perfusion. These local responses are further regulated by autacoids, hormones, and the autonomic nervous system (ANS).

Knowing the values of the main variables determining BP, one can develop the formula for BP. Based on these assumptions, arterial BP can be described as the product of cardiac output (CO) and total peripheral resistance (TPR): BP = CO × TPR. Unlike fluid flow in a pipe, the cardiovascular system is regulated by other systems, such as ANS which, among other things, regulates heart rate (HR) and SV. Thus, CO is the product of SV and HR:

$$\mathrm{BP}=\mathrm{SV}\times \mathrm{HR}\times \mathrm{TPR}$$

Calculation of TPR from this formula applies to non-pulsatile flow, and in the case of the circulatory system, the term mean arterial pressure (MAP) should be used instead.

$$\mathrm{MAP}=\mathrm{SV}\times \mathrm{HR}\times \mathrm{TPR};\;\mathrm{SV}\times \mathrm{HR}=\mathrm{CO};\;\mathrm{MAP}=\mathrm{CO}\times \mathrm{TPR}$$

According to the Hagen–Poiseuille law, the flow depends mainly on the radius of resistive vessels:

$$\mathrm{Flow}=\frac{\pi r4\Delta p}{8\eta l}$$

where

*r*: diameter of the vessel
Δ*p*: difference of pressures at the end of the vessel
*η*: viscosity coefficient
*l*: length of the vessel

The above-described relationships were already known in the nineteenth century and were described in works on the physiology of the cardiovascular system, while the importance of sodium as a factor determining volemia was noted already in the mid-nineteenth century by Carl Ludwig (Ludwig C (1885) Manuscripts of lecture notes, 1869–1870. In: Brunton TL, Williams FH (eds) A Textbook of Pharmacology, Therapeutics and Materia Medica. Lea Brothers and Co, Philadelphia, PA, p 503).

### Page’s mosaic theory of arterial hypertension

**Fig. 2:**
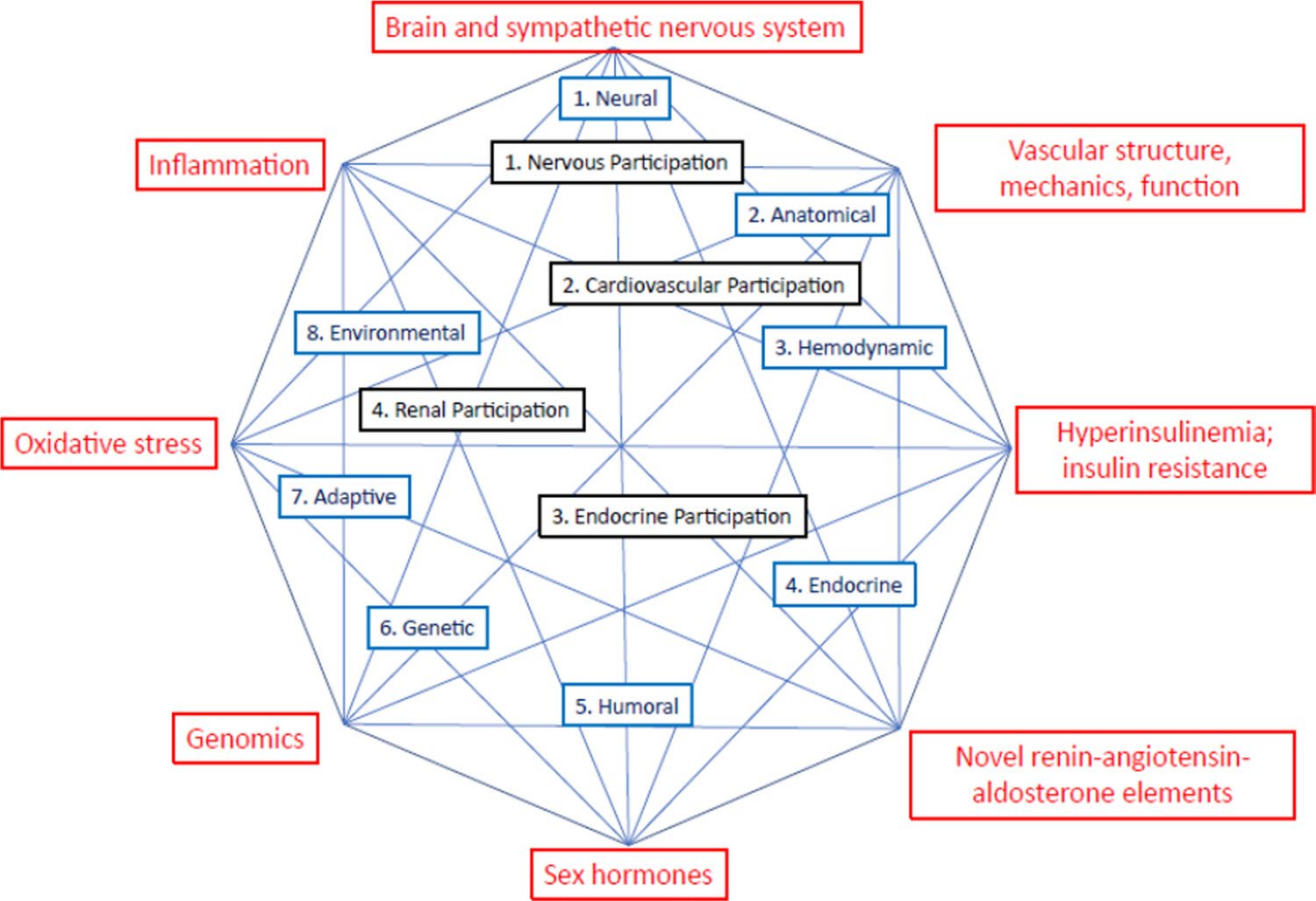
Proposed revised Page’s mosaic. The black elements are principle elements from 1949, and the 8 blue components are from a revised model from 1982. The red components are from a model proposed in 2020. Reprinted with permission from Touyz R et al. (2020) Can J Cardiol [3]

In the fifth decade of the twentieth century, in the face of an increasing amount of data, including experimental studies on animals, Irving Page proposed the first full theory of the pathogenesis of arterial hypertension, known as Page’s mosaic [1]. Over time, Page’s mosaic pattern began to be modified to include new mechanisms affecting the main determinants of BP, and some new nodes were added, while some old nodes were replaced by new ones, including those based on genomic research [2]. Recently, a new revised scheme of Page’s mosaic has been proposed (Fig. 2) [3].

It has been shown that the clinical-laboratory phenotype of children and adolescents with PH represents a set of pathophysiologically related disorders and corresponds to the main pathophysiological nodes of Page’s mosaic [4] (Table 1). The following sections of the article describe the main pathophysiological elements of primary hypertension (PH) related to Page’s mosaic nodes, based on studies in the general pediatric population and in children with primary hypertension.

**Table 1:** Clinical and laboratory phenotype of the child and adolescent with primary hypertension

**Clinical features**
Disturbed body composition – adiposity, visceral obesity [5, 6]
Accelerated biological maturation [7, 8]
Signs of hyperkinetic left ventricle and sympathetic drive [9–12]
**Laboratory features**
Metabolic abnormalities typical of metabolic syndrome [13–17]
Tendency to higher serum uric acid [18, 19]
Activation of innate and adaptive immunity [20–25]
Disturbed intestinal microbiota [26, 27]
Oxidative stress [28, 29]

## Volume or peripheral resistance — what matters in the pathophysiology of primary hypertension?

The problem of what is more important — volume or resistance — has been debated as early as 100 years ago by giants of physiology and cardiology. Hayasaka found that in patients with malignant hypertension and nephrogenic hypertension, as in the case of acute glomerulonephritis, volume expressed as CO was significantly increased in comparison with normotensive controls [30]. In contrast, the study by Burwell and Smith did not show any difference in CO in patients with arterial hypertension [31]. In 1936, Prinzmetal and Wilson gave indirect evidence that adults with PH showed signs of increased TPR and suggested that it was functional and potentially reversible, rather than permanent [32].

In the next few decades, two main theories on pathogenesis of PH were developed. One, described by Arthur Guyton, focused on the volume factor and kidney excretory function [33]. Guyton et al. induced arterial hypertension in laboratory animals (dogs) with reduced kidney mass and subjected to NaCl load. According to the experimental data of Guyton et al., an increase in volume caused by NaCl load causes an increase in CO and then a slow increase in TPR. Thus, salt-sensitive (SS) individuals require a higher BP to excrete the same amount of salt. In this model, the kidney defect causing salt retention is the main factor leading to BP elevation. Guyton’s mechanistic model has been developed in greater detail and involves many mechanisms regulating BP such as baroreceptors and chemoreceptors, stress, and renin-angiotensin system (RAS). A clinical example of the validity of Guyton’s theory is volume- and salt-dependent hypertension in patients with acute kidney injury or advanced chronic kidney disease.

The second hypothesis presented by Folkow states that a structural remodeling of resistance vessels is a cause of sustained hypertension [34]. Folkow stated that in PH — as well as in animal models of arterial hypertension, such as in spontaneously hypertensive rats or in renovascular hypertension — the problem lies in vascular remodeling of resistance vessels. It leads to a greater increase in resistance at the same degree of smooth muscle shortening in hypertensive subjects compared to normotensives. From a biophysical point of view, increased BP will increase circumferential wall stress. As circumferential wall stress depends on pressure, internal arterial radius, and wall thickness, in order to decrease or normalize circumferential wall stress, the arterial radius must be decreased or the wall thickness increased. Different arteries react in different ways. Large conduit arteries, such as the aorta or common carotid arteries, decrease wall stress by increasing wall thickness. It can be clinically observed as increased carotid intima-media thickness (cIMT), which is well documented in pediatric studies. In contrast, a different reaction dominates in small resistant arteries: it is a decrease in radius which not only decreases wall stress but also controls tissue perfusion.

## Salt sensitivity and salt resistance

In fact, both volumetric and resistance mechanisms work together, and there is ample evidence of a close relationship between SS and increased TPR.

Although the relationship between salt and pressure was described as early as the nineteenth century, the significance of the SS phenomenon in clinical trials has been described by Kawasaki et al., followed by Weinberger et al., and at the general population level in the INTERSALT study which studied subjects aged 20–59 [35–37]. On the other hand, a large part of the general population does not respond to an increased supply of NaCl with an increase in BP, and some patients with PH do not respond to a reduction in salt intake and are salt resistant (SR). The definitions of SS and SR have changed over time. In the study by Weinberger et al., SS was defined as decrease of MAP by 10 mmHg after salt depletion; decrease of MAP by less than 5 mmHg was defined as SR [36]. The currently used protocols for assessing SS and SR in adults have been described in the American Heart Association’s statement on SS [38]. For children and adolescents, it is proposed to use definitions described by Kurtz et al., who recommended defining SS as ≥ 3–5 mmHg change in MAP for normotensives and ≥ 8–10 mmHg for hypertensives [39, 40]. It is estimated that nearly 50% of hypertensive adults are SS compared to 25% of normotensive adults. However, one should be aware that SS increases with age and accompanying disorders. Importantly, SS is more common among women and Black subjects [41, 42].

Further evidence of the importance of both salt and increased TPR in the pathogenesis of PH has been provided by studies on monogenic hypertension [39]. It is worth noting, however, that only one of the 13 forms of monogenic hypertension is caused by a primary increase in TPR and smooth muscle hypertrophy of the resistance vessels. The other 12 forms of monogenic hypertension are salt dependent and present as low-renin hypertension; 5 of them are caused by primary hyperaldosteronism, 5 by mineralocorticoid action caused by other steroid hormones or hypersensitive mineralocorticoid receptor (MR), and 2 by defects in sodium channels: epithelial sodium channel (ENaC) and sodium chloride cotransporter (NCC). Initially, according to Guyton’s hypothesis, it was believed that the increase in BP caused by the NaCl load is a consequence of hypervolemia, which suppresses renin generation, causes increased CO, and then, after the increase in BP, natriuresis occurs and a new, higher BP level necessary to excrete the salt load is established. Subjects who were able to excrete a load of salt at lower pressures were not expected to develop hypertension, in contrast to those who showed impaired natriuresis and needed higher BP to excrete NaCl load. However, a more thorough analysis of the generation of hypertension in salt-dependent forms of monogenic hypertension indicates that the increase in CO is not greater than in normotensive subjects, and the main disorder is an inadequate reduction in TPR compared to normotensive subjects who were loaded with NaCl. Based on these observations, a new model of generation of SS hypertension was proposed, in which NaCl load disrupts the mechanism of reducing TPR in SS and/or low-renin forms of monogenic hypertension [39].

The mechanistic explanation of the mechanism of SS and inadequate reaction of resistive vessels to salt loading can be explained by organ and tissue expression of MR and ENaC. MR is expressed not only in distal tubules but also in the brain, colon, endothelium, and adipose tissue; ENaC is expressed in distal tubules, the brain, lungs, exocrine glands, dendritic cells, and arterial endothelium as endothelial sodium channel (EnNaC) [43–47]. In the case of familial hyperkalemic hypertension (FHH) in which the activity of the NCC is increased, all of the genes that cause FHH relate to genes encoding proteins that regulate the activity of the NCC, but not to the NCC itself. It is suggested that these mutant proteins are involved not only in the control of NCC activity but also in the contraction of resistance vessels. It was found in a mouse model for Liddle’s syndrome that increased activity of brain ENaC causes hypertension, activation of EnNaC stiffens arterial walls and inhibition of ENaC increases endothelial nitric oxide synthase in rat mesenteric artery. Similarly, activation of endothelial MR in resistive vessels activates EnNaC. The importance of these effects of MR and EnNaC has been confirmed in clinical trials that have shown a reduction in arterial stiffness expressed as pulse wave velocity (PWV) in patients treated with MR antagonists [48].

An additional mechanism explaining SS and blood pressure lowering effects of potassium intake as well as the importance of NCC and ENaC in the regulation of renal sodium retention is the potassium switch hypothesis [49]. Potassium switch hypothesis is based on an analysis of alterations caused by WNK4 and SPAK kinase mutations in FHH. Physiologically, WNK4 and SPAK, which regulate the function of thiazide-sensitive NCC, are activated by potassium deficiency. It leads to increased sodium reabsorption by NCC and lower sodium delivery to distal parts of nephron and increased reabsorption of potassium. In the case of sufficient potassium intake, NCC action is suppressed and potassium secretion in aldosterone-sensitive nephron segments is facilitated.

Another factor in the regulation of SS is the buffering role of the skin, subcutaneous space and interstitial tissue glycosaminoglycans (GAG) [50]. Interstitium, which consist of a fluid phase, collagenous matrix and GAGs, can store up to 30% of total body sodium. Greater capacity of interstitium GAGs to store sodium may decrease SS. The lower number of GAGs in women may explain higher prevalence of SS in women.

Another aspect of the wide expression of ENaC, MR, and proteins regulating NCC, and the role of skin interstitial sodium storage, is the activation of the sympathetic nervous system (SNS) and the immune system, which is described later in this article.

Most clinical and population-based studies on SS are studies in adults. A review of pediatric research on this topic has recently been presented in detail by Hanevold [51]. In short, pediatric studies showed that SS is evident in children with obesity, diabetes (both type 1 and type 2), born prematurely and as small for gestational age (SGA), and it is more often found in African-American children and adolescents [52–55]. Falkner et al. found that after oral salt load for 2 weeks in adolescents and young adults (18–23 years of age), 37% of non-Hispanic Black subjects were SS compared to 18% of white subjects. Importantly, the comparison of studies involving children and young adults of different ages indicates that the frequency of SS increases with age, at least among non-Hispanic Black subjects [53]. However, there are no studies analyzing the role of sodium buffering by interstitium in children with PH.

The feature of SS was used in the selection of antihypertensive drugs. Laragh proposed to use renin profiling as a guide for choosing antihypertensive drugs — diuretics or vasodilators [56]. Recently, Rayner and Spence proposed a similar approach to choosing antihypertensive medication for Black hypertensive subjects [57].

## Autonomic nervous system abnormalities in primary hypertension

The role of both SNS and the parasympathetic part of ANS is viewed both as a physiological mechanism of fast control of BP fluctuations and as an important system in long-term BP control, playing a significant role in the development and maintenance of PH. There are multiple levels of SNS which include (1) regulatory centers in the central nervous system (CNS); (2) ganglionic transmission; (3) release, clearance, and reuptake of neurotransmitters; (4) adrenergic receptors; and (5) post-receptor pathways and tissue and organ responsiveness. Thus, to assess different parts of SNS directly, the norepinephrine spillover technique and the microneurographic recording of efferent postganglionic muscle sympathetic activity have been developed. However, due to their invasive nature, these techniques have not been used in pediatric studies. There are large amounts of data, both from experimental studies and clinical observations in adults and children with PH, that says that altered SNS activity is associated with the generation and maintenance of PH, visceral obesity, metabolic abnormalities, and immune phenomena typically observed in pediatric and adult patients with PH [58]. Despite its limitations, HR is used as a surrogate marker of SNS activation. Already the first reports from the Framingham Study indicated that a faster HR preceded the development of hypertension [59]. Julius et al. in the Tecumseh study showed that there was a bi-directional relationship between faster HR and higher systolic blood pressure (SBP), and faster HR and adiposity: the faster heart rate at the age of 8, the greater subscapular skinfold at the age of 16 and 30, and the higher SBP at the age of 8, the greater adiposity and faster HR at the age of 16 and 30 [60]. In the next decades, numerous studies in adults showed that PH is associated with increased muscle sympathetic activity and increased renal and cardiac noradrenaline spillover. Increased SNS activation has been documented in obese adults and was even greater in obese-hypertensive subjects compared to obese-normotensive subjects. It was also found that metabolic syndrome (MS) was associated with increased SNS activity in adults [61].

One of the first pediatric reports analyzing associations between SNS and BP are from the Bogalusa Heart Study in which an increased HR correlated with higher BP and skinfold thickness as a marker of adiposity [62]. Furthermore, a “double product” — i.e., HR multiplied by MAP — was significantly elevated in obese children, which would indicate that obese children suffer from greater adrenergic stimulation. It is important to note that both HR and the double product values were greater in white boys than in African-American boys. In subsequent reports from this prospective study, it was observed that the MAP and HR double product changed in adulthood in a crossover manner indicating a greater SNS activation occurring in Black subjects reaching adulthood [63]. This effect of age and race was also observed in another substudy of the Bogalusa Heart Study, in which Fourier analysis of the Holter ECG showed significantly a higher ratio of low frequency (LF) to high frequency (HF) activity — a marker of sympathetic to parasympathetic balance — in white children compared to Black children [64]. Increased SNS activity may be driven by impaired baroreflex and/or due to central SNS activation. Clinical studies in hypertensive children using indirect methods of assessment of ANS activity have shown that both mechanisms play a role. Genovesi et al., analyzing the sympathovagal function by using markers of oscillatory (LF, HF) and nonlinear modulation of the sinoatrial node and baroreflex slope, found that both pre-hypertensive and hypertensive children had significant baroreflex impairment compared to control subjects [65, 66]. The study analyzing BP and HR rhythms (Fourier analysis of 24 h ABPM recordings) as indirect markers of SNS activity in normotensive, white coat hypertensive (WCH) and PH children found a higher prevalence of shorter 12 h ultradian rhythms in PH and WCH children, and both groups had reduced BP amplitudes and delayed BP acrophases compared to normotensive children, which indicates prolonged SNS activation during the 24-h rhythm [9]. Moreover, a decrease in the amount of visceral fat assessed by magnetic resonance imaging was associated with a normalization of disturbed BP rhythms [10].

Since one of the main phenotypic features of the hypertensive child/adolescent is visceral obesity and disturbed body composition, greater SNS activity in adipose subjects may be an important pathophysiological factor in the generation and maintenance of PH in childhood. However, not all overweight and obese subjects are hypertensive. It is well documented that the afferent signal transmitted by leptin to CNS must be intact to trigger SNS activation. The activation of SNS by leptin is due to the release of melanocortin, which acts on melanocortin 4 receptor (*MC4R*) and causes SNS activation. Thus, both leptin mutations and *MC4R* variants do not show signs of SNS activation expressed as faster HR, elevated BP or elevated urinary norepinephrine excretion compared to obese patients without *MC4R* mutation [67]. It was also found that patients with an *MC4R* variant had lower muscle sympathetic nerve activity after 30 s of apnea than non-carriers [68]. In contrast, adolescent carriers of the *FTO* gene risk variant showed higher BP and signs of SNS activation expressed as greater increase in SBP during mental stress and greater adrenergic drive as assessed by power spectral analysis of diastolic BP [69]. These observations are in line with Landsberg’s hypothesis, describing the phenomenon of sympathetic activation in obesity. Landsberg stated that the SNS activation in obesity is a metabolic reflex, which causes dissipation of excess energy, and clinical markers of this reflex are increased HR and elevated BP. However, chronic activation of SNS decreases thermogenesis but unopposed leads to hypertension [70].

## The role of disturbed body composition

More detailed analyses indicate that it is not obesity but rather disturbed body composition with visceral adipose tissue distribution and male sex that are important in generating SNS activity. Studies of 324 adolescents of French-Canadian origin found that visceral fat deposition correlated both with BP elevation and increased SNS drive only in boys, but not in girls. In addition, higher androgen receptor activity was associated not only with higher BP (the difference between high-activity receptor carriers and intermediate- and low-activity carriers was from 1.8 up to 8 mm Hg) but also with increased BP during mathematical stress. Moreover, boys with the high-activity receptor form had a greater amount of visceral fat and higher activity of sympathetic tone, as assessed by power spectral analysis of diastolic BP [69, 71, 72]. These associations have not been found in girls. In another study of this group, it was found that it is visceral fat that determines BP elevation in boys and total body fat in girls [72]. The role of visceral fat in SNS activation expressed as disturbed BP rhythmicity was also evidenced in a prospective study, in which normalization of disturbed BP rhythms in hypertensive adolescents was associated not with a decrease in BP decrease but with a decrease in the amount of visceral fat [10].

## Hemodynamics of primary hypertension in children and adolescents

Clinical studies showed that typical the hemodynamic phenotype of childhood hypertension is isolated systolic hypertension and elevated HR, which dominates both among obese and non-obese hypertensive adolescents [73, 74]. Such a pattern suggests hyperkinetic function of LV. More detailed studies with the use of invasive assessment of LV function revealed that LV and TPR change in the course of PH. Results of the Bergen study, in which hemodynamics was assessed with an invasive method, showed that older adolescents and young adults with PH aged 17 to 29 years evolved from hyperkinetic heart pattern with faster HR, increased CO and normal TPR to normal CO, and increased TPR in 2 decades [75]. This study also showed that the longer the duration of hypertension, the greater the TPR. The ENIGMA study including subjects with a mean age of 30 years showed sex-related differences in hemodynamic pattern: hypertensive young adult males demonstrated elevated CO and normal TPR and hypertensive young females demonstrated lesser increase of CO, but with significantly greater TPR [76]. A similar pattern was found in a younger (mean age of 15 years) cohort of adolescents with PH who demonstrated greater CO, SV, and slightly lower TPR across BP ranges, and the same was found after the exclusion of obese subjects [77]. A more detailed study analyzing kinetics of LV contraction in adolescents with PH showed faster ejection flow from LV, increased HR, and CO indicating hyperkinetic LV function [11]. Recent meta-analyses of pediatric studies showed that children with PH are characterized by hyperkinetic LV expressed as increased fractional shortening, ejection fraction, CO, and normal TPR [12, 78]. Moreover, there was an evolution from increased CO at a younger age to increased TPR at an older age. What is more, the meta-analysis showed that the more severe the PH, the greater the TPR. These results indicate that hemodynamics of PH evolves from a “cardiac” phenotype presented as a hyperkinetic LV to a “vascular” phenotype with less pronounced elevation or even normalization of CO and increased TPR [12].

## Vascular remodeling in childhood hypertension

One of the nodes of Page’s mosaic are vascular factors, which means disturbed structure and function of both large and small resistive arteries. Due to the development of non-invasive diagnostic methods, it is possible to assess both macrocirculation and microcirculation in children with arterial hypertension. Arterial wall remodeling in PH is associated with increased arterial stiffness as well as with both faster PWV and higher pulse pressure. Hypertensive remodeling of carotid arteries expressed as increased cIMT is observed in adolescents with PH already at diagnosis and increases with the BP status increasing from normotension to stage 2 hypertension [79]. There is also strong evidence that cIMT is determined by metabolic abnormalities typical of MS, oxidative stress, and immune abnormalities (see below). On the molecular level, hypertensive arterial remodeling is associated with disturbed matrix metalloproteinases (MMP) and their tissue inhibitors (TIMP) balance. Hypertensive adolescents were found to have disturbed serum concentrations of *MMP9* and *TIMP1* and their gene expression in peripheral blood leucocytes (PBL) [80, 81]. It is important to note that these associations were evident in boys, but not in girls. These and other observations were the basis for presenting the hypothesis of earlier biological aging not only of the arteries, but as a systemic phenomenon involving the vascular system, metabolism, and the immune and nervous systems [4].

With regard to microcirculation, there is much less data from pediatric studies. Retinal vessels are the site of assessment of microcirculation in vivo. Their surface and diameter is an anatomical equivalent of TPR. The results of the Hanssen group indicate that already in children from the general population at the age of 7, it is possible to observe a relationship between the smaller diameter of the retinal arteries and BP, as well as a negative correlation between the narrowing of retinal arteries and BMI and lower physical fitness [82]. In addition, a prospective study showed that both retinal artery narrowing was a predictor of higher blood pressure at the age of 11 and higher blood pressure at the age of 7 was a predictor of retinal vasoconstriction at the age of 11 [83]. As with other pathophysiological factors, microcirculation structure and function seem to be associated with race. In a population-based study of European and South African children, Black South African children had wider retinal veins and narrower arterioles compared to European Caucasian children, regardless of obesity [84]. Microcirculation and macrocirculation structure and function are closely related. Studies in 7-year-old children from the general population found that lower diameters of retinal arteries are associated with carotid-femoral PWV after 4 years [83]. In a cross-sectional study of adolescents with PH, it was found that the avascular area of the retina (a surrogate marker of structural decrease of microcirculation) was determined by cIMT and central SBP [85].

The results of the above-cited studies show that both macrocirculation remodeling and microcirculation remodeling are present already at diagnosis of PH, and in children from the general, healthy population, it correlates with BP values and can predict future BP values. However, as with other determinants of PH, it is still not known which comes first: arterial remodeling or increased blood pressure.

## Metabolic abnormalities and oxidative stress

The next node in Page’s mosaic is the metabolic factor associated with arterial hypertension. Children and adolescents with PH are typically exposed to metabolic abnormalities characteristic of MS with hyperinsulinemia and insulin resistance (IR), higher uric acid levels, and oxidative stress (SOX) [4]. Their extent in childhood PH is closely associated with altered body composition and visceral fat. The relationship between serum insulin concentrations, IR, glycemia, and BP status is already present at the age of 4, progresses over time until young adulthood, and is associated with measures of disturbed body composition [13]. Moreover, higher insulin levels and IR at the age of 13 predicted the elevation of BP and development of dyslipidemia at the age of 16, independently of BMI [14]. Another typical metabolic abnormality for PH is the tendency to have higher uric acid levels. Serum uric acid levels, even in the upper normal range (> 5.5 mg/dl), distinguished adolescents with PH from those with WCH and secondary hypertension [18]. Moreover, allopurinol, used in adolescents with PH and with serum uric acid levels above 6 mg/dl, lowered both uric acid levels and BP [19].

SOX is both a non-specific marker for metabolic abnormalities typical of MS and their consequence. There are only a few clinical studies in children with PH in which SOX markers were analyzed. However, the results of these studies indicate that SOX is not only higher in hypertensive children compared to their normotensive peers, but it is also closely associated with PH, irrespective of BMI, and correlates with hypertension severity, hypertension-mediated organ damage (HMOD), visceral obesity markers, and other metabolic and immunologic abnormalities [28]. SOX expressed by serum concentration of thiobarbituric acid substances was found to be associated with diastolic blood pressure and arterial stiffness in prepubertal, healthy, normotensive boys [29]. Moreover, a prospective study showed that normalization of SOX was associated with regression of HMOD and normalization of metabolic abnormalities and immune activity [28].

There is also a relation between adipocytokines and the immune system, especially adiponectin, which is an anti-inflammatory, and insulin-sensitizing cytokine. Adipocytokines, such as adiponectin and leptin, combine the effects of abnormal body composition, metabolic disturbances, and immune abnormalities. An analysis of 113 children with untreated PH who were examined at the time of diagnosis showed that serum levels of adiponectin correlated negatively with cIMT, and adiponectin levels were found to be the main determinant of cIMT [15]. Adiponectin action is mediated by specific receptors on target cells, including on cells within the immune system. PBL of hypertensive children express adiponectin receptors. Those receptors inversely correlated with serum adiponectin levels, irrespective of BMI, and correlated with hypertension severity — the more severe the hypertension, the greater the expression of adiponectin receptors on PBL [20].

## Immune abnormalities in childhood hypertension

Immune disorders were not included in Page’s original mosaic. However, the results of experimental studies since the 1960s and clinical observations pointed to the immune system as another node in Page’s modified mosaic. Non-specific activation of the innate immune system is well described in both adults and children with PH and correlates with HMOD, hypertension severity, and metabolic abnormalities [reviewed in 86]. However, there are large amounts of data indicating that the adaptive T cell-dependent system is crucial for maintaining hypertension. This was documented by the transfer of hypertension using lymph node cells from hypertensive to normotensive rats, evidence that an intact thymus was necessary for maintaining elevated BP in hypertension-prone rats and mice, the establishment of the role of thymus-derived lymphocytes in the chronic stage of experimental hypertension but not in the early phases of experimental hypertension, and the discovery that an athymic hypertension-prone New Zealand Black strain of mice did not develop hypertension. Other studies have shown RAG − / − mice that do not have T-lymphocytes and do not have elevated BP during angiotensin 2 (AT2) infusions, but the adoptive transfer of T cells restored sensitivity to AT2. It was also found that T regulatory cells (T regs), which limit the extent of the immune response, play a significant role in the pathogenesis of arterial hypertension within the experimental setting [reviewed in 86].

The pathogenetic link explaining the activation of the adaptive immune system with the maintenance of hypertension is the generation of autoantigens from the damaged arterial wall. Autoantigens are intracellular proteins and lipids which undergo oxidative modifications. Oxidized lipids link with proteins and form new peptides which are recognized as non-self by dendritic cells (DC). Experimental studies have shown that isolevuglandins, which are lipid oxidation products, and heat shock proteins, especially heat shock protein 70, are very immunogenic and are assumed to form neoantigens [87]. It is proposed that the first step to the transition to the chronic phase of hypertension is damage to arterial wall by hemodynamic insult accompanied by metabolic and innate immunity factors and activation of endothelial surface [88]. Long-lasting exposure of the arterial wall to hemodynamic and immuno-metabolic injury causes release of neoantigens, which starts the second phase of hypertensive disease with the activation of the adaptive immune system and the perpetuation of hypertension.

**Fig. 3:**
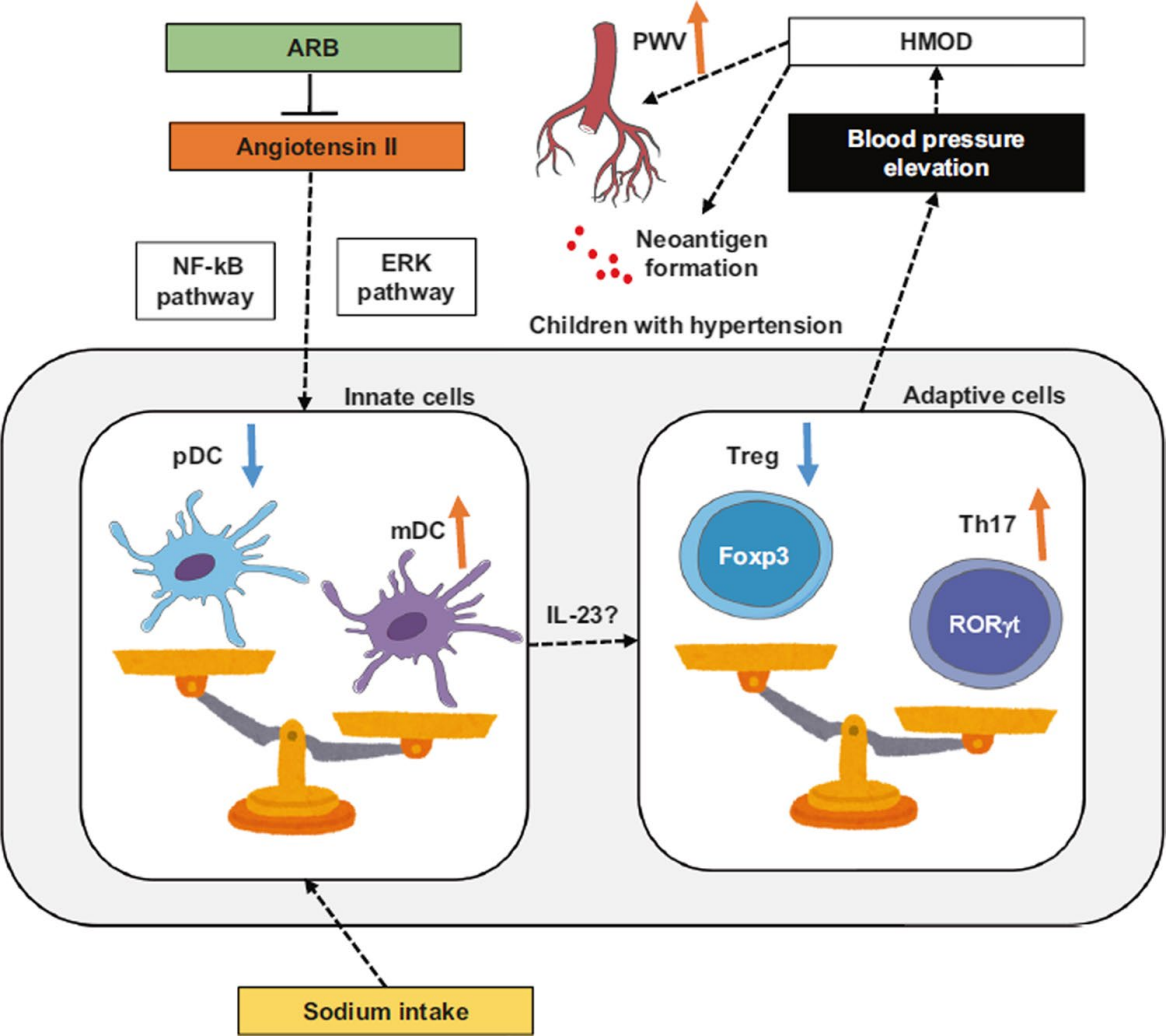
Imbalance of dendritic T cells in pathogenesis of arterial hypertension. ARB, angiotensin receptor blocker; DC, dendritic cells; ERK, extracellular signal regulated kinase; IL23, interleukin 23; mDC, myeloid dendritic cells; NF-kB, nuclear factor kappa B; pDC, plasmocytoid dendritic cells; Treg, T regulatory cells; Th17, T helper cells producing interleukin 17. Reproduced with permission from Higaki A, Modi M (2022) Hypertens Res [89]

Pediatric clinical studies published in the last 2 decades documented that compared to their normotensive peers, children with PH already at diagnosis showed significant activation of innate immunity with increased serum concentrations of not only hsCRP but also chemokines, which indicates endothelial activation. These markers significantly correlated with the BP status, HMOD including cIMT, and with metabolic abnormalities typical of MS [reviewed in 86]. PBL, the main cells of the immune system, cooperate with both RAS, SNS, and adipocytokines generated by adipose tissue. PBL in children with PH had a greater expression of adiponectin and RAS genes, and this pattern of gene expression normalized after 6 months with non-pharmacological treatment [21]. Immune cells control extracellular matrix remodeling by excretion of MMPs and their TIMPs. Untreated children with PH had significantly higher serum levels of *MMP-9* as well as disturbed patterns of MMPs and TIMPs secretion and gene expression compared to their normotensive peers [80]. *TIMP-1* serum concentrations correlated with markers of aortic stiffness, such as aortic pulse pressure and augmentation index, and an altered expression pattern of *MMP/TIMP* genes in PBL was associated with LVH, the criteria for MS, and visceral obesity [81]. Other studies revealed that hypertensive children compared to their normotensive peers show significant alterations of T cell distribution, more mature memory T cells, a lower percentage and number of T regs, and a shift towards an activated/memory phenotype of T regs [22, 23]. Moreover, the pattern of T cell subset distribution and the percentage of memory T regs were associated with LVH and markers of aortic stiffness, such as PWV and augmentation index [23]. In another study, adolescents with PH showed a significantly greater percentage of activated myeloid DC, and the expression of DC activation markers correlated with cfPWV [24]. It is important to note that children with WCH showed an intermediate DC phenotype between the normotensive and hypertensive phenotypes. DCs are antigen-presenting cells, and one of the sites of their residence is the skin and subcutaneous tissue. Since subcutaneous tissue is a store for sodium, it is suggested that Na may enter DCs via ENaC and is replaced through the action of the Na + /Ca2 + exchanger. The next step is Ca2 + influx, which stimulates protein kinase C (PKC), which in turn increases NADPH activity, driving the formation of isolevuglandins (Fig. 3) [89].

## Microbiome

The results of research on the intestinal microbiome published in recent decades indicate that hypertension is associated with intestinal dysbiosis [26]. In addition, disorders of the intestinal microbiome combine the main pathophysiological disorders described so far, such as activation of the immune system, presentation of neoantigens, SS, and obesity.

Pediatric studies mainly focus on obese children with obesity, type 2 diabetes, and chronic kidney disease [27, 90]. Experimental studies have shown that the composition of the microbiota depends not only on diet and calories but also on the salt consumed. In both humans and mice, high salt intake was associated with changes in the gut microbiome, reflecting an increase in Firmicutes, Proteobacteria, and Prevotella genus bacteria. These alterations were associated with higher blood pressure in humans. In mice, high salt intake caused vascular inflammation and hypertension in response to a subpressor dose of angiotensin II. In addition, it was found that high salt intake led to the formation of modified isolevuglandin proteins in DCs [91].

## Perinatal risk factors and perinatal programming of cardiometabolic disorders

Although hypertension in subjects born preterm or as SGA appears to be a separate form of hypertensive disease, prematurity and SGA-associated disorders exemplify the accumulation of all previously described pathophysiological phenomena in PH, such as SS, insulin resistance and MS, visceral obesity, greater adrenergic drive, intestinal dysbiosis, and activation of the immune system. Therefore, perinatal factors, prematurity, and SGA not only summarize all main pathophysiological nodes of Page’s mosaic but additionally modulate all of the major pathophysiological abnormalities in PH described above.

There is significant evidence indicating that SS in premature children and adolescents is associated with lower kidney mass, which is consistent with Brenner’s theory. Simonetti et al. found that 11-year-old children born prematurely or as SGA were SS in 37% and 47%, respectively [55]. Moreover, SS correlated negatively with kidney length. In the study by Raaijmakers et al., SS ex-preterm 11-year-old adolescents also had lower plasma renin activity, kidney length, and eGFR [92]. The risk of cardiometabolic abnormalities is greater in those subjects who were born as SGA. Lurbe et al. documented in a prospective study that children born as SGA had higher insulin levels at the age of 5, even if they were not obese, and at age 10, those with higher insulin levels also had higher BP and were exposed to other MS abnormalities as well as having higher uric acid levels [16, 17].

## Conclusions

**Fig. 4:**
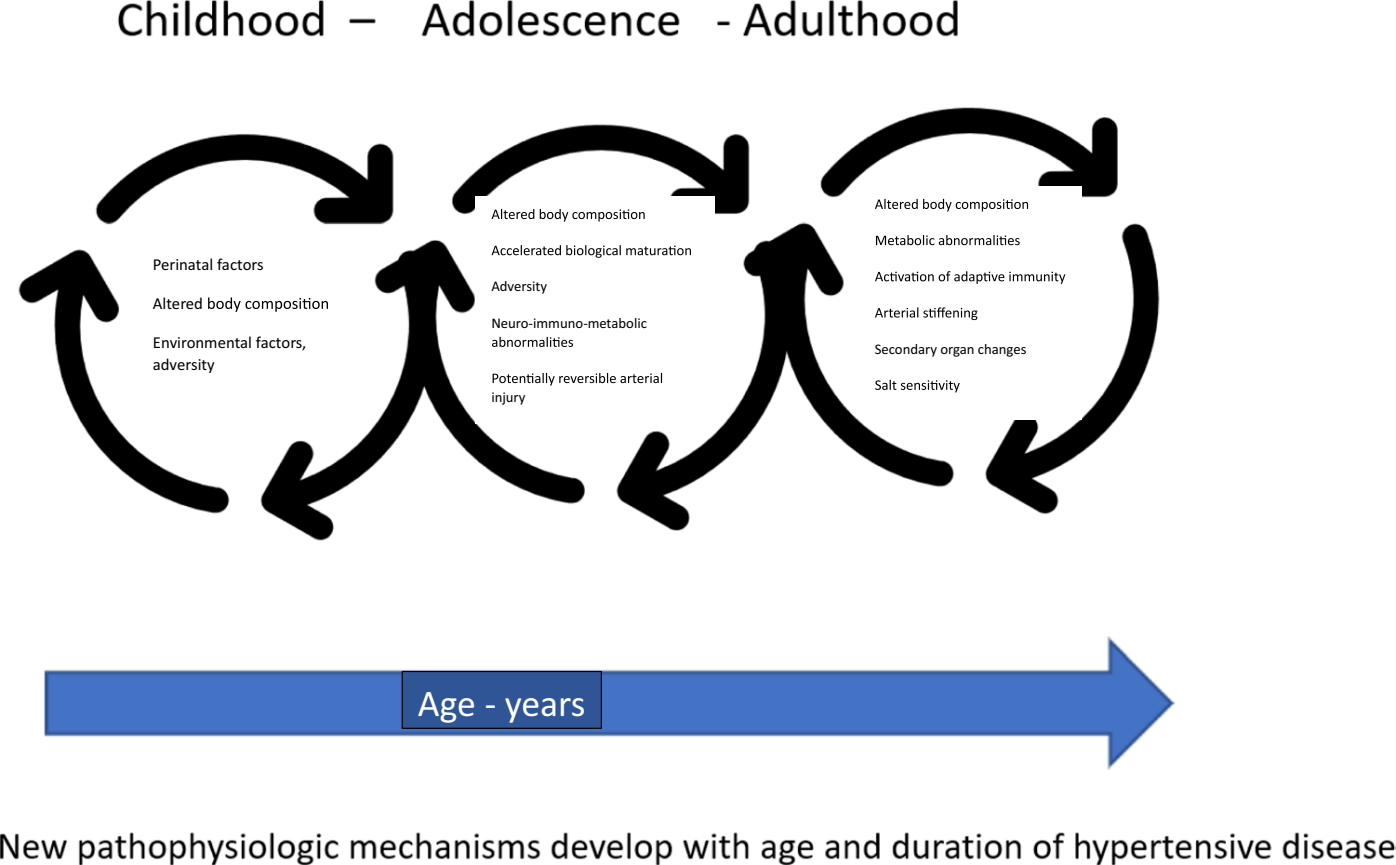
Arterial hypertension development. Arterial hypertension develops gradually. Different mechanisms are involved in the increase in blood pressure at different stages of biological development. With age and the persistence of the original disorders, changes such as disturbed body composition, adaptive vascular changes, damage to the microcirculation, and immune activation gradually generate new, irreversible arterial injury with increased stiffness resulting in the perpetuation of hypertension. The evolution of these lesions is modulated by genetic susceptibility

Page’s mosaic, presented for the first time almost 70 years ago, is still, after modifications related to obtaining new research results, a model describing the multifactorial nature of PH. Observations made in recent decades have proven not only the phenomenon of early development of PH, but also its relationship with disorders in the course of pregnancy, perinatal period, environmental influences, and adversity in early childhood. The clinical and laboratory phenotype, the evolution of the hemodynamics of PH, and events at the cellular level, including inflammatory response, indicate that the pathophysiological phenomena in PH evolve with the course of the disease. The importance of individual pathophysiological factors changes over time (Fig. 4). Hypertensive remodeling of the arteries, which is initially a physiological response to increased arterial wall tension, over time leads to increased arterial stiffness and becomes the main factor behind the isolated increase in SBP. Similarly, with the duration of the disease, and thus with age, phenomena such as SS, hyperkinetic LV, or increased TPR gain a different clinical significance. With age, there is an increase in exposure to behavioral and environmental risk factors, such as reduced physical activity and obesity, smoking, risk of type 2 diabetes, and stress. Therefore, it seems that the next node in the pathophysiological mosaic of PH should be age. Another research challenge remains the importance of sex. In contrast to the adult population, in the pediatric population, PH is 3–4 times more likely to affect boys. Differences in the incidence of PH between both sexes disappear only in the 5th decade of life. However, even with all nodes of Page’s mosaic, all pathophysiological factors act on the basic blood pressure variables, and the formula CO × TPR = BP is still valid.
